# Comparison of in vivo antibacterial and antithrombotic activities of two types of pulmonary artery catheters in pig

**DOI:** 10.1186/s40824-017-0109-3

**Published:** 2017-11-15

**Authors:** Jung Wook Han, Yeon Soo Shin, Jung Ju Kim, Ho Sung Son

**Affiliations:** 1 Department of Thoracic and Cardiovascular Surgery, Korea University Medical Center, #73, Inchon-Ro, Sungbuk-Gu, Seoul, 136-705 South Korea; 2 Korea Artificial Organ Center, Korea University, #73, Inchon-Ro, Sungbuk-Gu, Seoul, 136-705 South Korea

**Keywords:** Pulmonary artery catheter, Infection, Thrombosis, Pig

## Abstract

**Background:**

During pulmonary artery catheter (PAC) implantation, inaccurate measurements of hemodynamic parameters due to infection or thrombosis of PAC can result in severe complications.

**Method:**

In order to develop a new PAC material, we evaluated the antibacterial and antithrombotic activities of the two types of PAC (Swan Ganz catheter and prototype catheter) in 14 pigs.

**Results:**

In the 3-day group, bacterial infection rate was not different between the two types of PAC. In the 7-day group, bacterial infection rate of the prototype catheter was twice as elevated as that of the Swan-Ganz catheter. In the 3-day group, thrombus formation rate of the prototype catheter was twice as elevated as that of the Swan-Ganz catheter. In the 7-day group, thrombus formation rate was the same for the two types of PAC.

**Conclusion:**

Here, we report an experimental pig model that confirms differences in antibacterial and antithrombotic activities.

## Background

Pulmonary artery catheter (PAC) has been widely used in critical care since the 1970s. It is reported that 1.5 million PACs are used annually in the United States to monitor hemodynamic status of critically ill patients [1]. It plays an important role in assessing the patient’s condition in the intensive care unit or operating room, establishing a diagnosis of underlying pathology and deciding on the treatment plan by measuring hemodynamic parameters, such as cardiac output, mixed venous oxygen saturation and intra-cardiac pressures [2–6].

PAC consists of blood compatible catheter, temperature sensor and monitoring system. Biofilm formation due to infection or thrombosis near temperature sensor has been reported to cause serious problems [7–9]. As a result, the sensitivity of sensor was also reduced [10]. Currently, most cardiovascular medical devices are coated with lubricant, antithrombotic or antimicrobial agents. But there are no products that fundamentally solved serious side effects caused by prolonged implantation.

We compared the antibacterial and antithrombotic activities of the most commonly used PAC, Swan-Ganz catheter (CCCO Combo catheter: Vigilance II, Edwards® Lifesciences, California, USA), with those of the prototype catheter in a pig model in an effort to develop a new catheter material.

## Materials and methods

Main difference between the two types of PAC is antithrombogenecity. Prototype catheter improved antithrombotic activity through a newly developed heparin coating procedure. Prototype catheter was composed of 72% Pellethane® 2363-55D (CAS # 37383–28-1, Compounding Solutions, Lewiston, Maine, USA), 20% Barium Sulfate (CAS # 7727–43-7, Compounding Solutions, Lewiston, Maine, USA) and 6% AD85H-M® (Antimicrobial agent, Compounding Solutions, Lewiston, Maine, USA). The shape of prototype catheter was similar to that of Swan-Ganz catheter.

Because several studies have documented that the cardiovascular system in pig is similar to that in humans, when compared with other animal models [11–13], we experimented with 14 female pigs. The subjects of this study were female Yorkshire Swine pigs weighing 45 ± 5 kg. The animals used in our experiments came from farms (XP bio® or Optipharm®, Cheongju, Korea) licensed by the Ministry of Food and Drug Safety, Republic of Korea. Pigs were moved from this farm to the Laboratory Animal Research Center Korea University College of Medicine 7 days before experiment and were housed in individual cages under controlled environments until experiment.

**Fig. 1:**
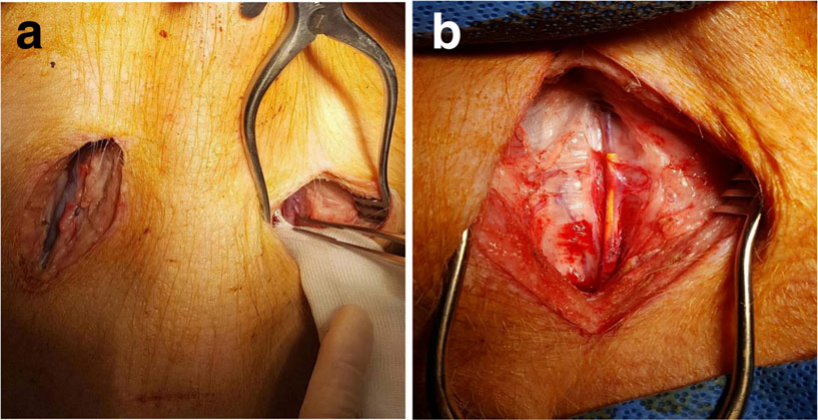
Operative findings. **a** Exposure of both external jugular veins, **b** External jugular vein containing a catheter fragment

Bilateral external jugular veins were used to reduce errors due to individual difference (Fig. 1a). Experiments were conducted as follows. Prophylactic antibiotics (cefazolin 1 g, Chong Kun Dang®^,^ Seoul, Korea) were injected intravenously 1 h before skin incision. General endotracheal anesthesia was induced with thiopental sodium 5–10 mg/kg and vecuronium bromide 0.1 mg/kg. Bilateral external jugular veins were exposed under sterile condition. Before jugular vein clamping, unfractionated heparin 50 IU/kg was injected intravenously. The fragment of catheter cut into 3 cm length was inserted into the vein via a tiny slit and were held to the vein with 6–0 prolene suture, which was used to close the venotomy (Fig. 1b). After hemostasis, the surgical wound was closed layer by layer. Low molecular weight heparin (LMWH, Clexane, Sanofi-Aventis®^,^ Paris, France) at a dose of 1 mg/kg was injected subcutaneously every day until the catheters were removed. Three days later, 7 pigs underwent reoperation to remove the catheters. External jugular vein containing the catheter was resected into 4 cm length. We were careful not to disturb any thrombus that may have been present. The remaining 7 pigs underwent reoperation 7 days later. At the end of the operation, all pigs were euthanized by intravenous injection of potassium chloride 2 mEq/kg.

## Results and discussion

Catheter tip culture was performed, and the culture was subsequently incubated for 48 h to evaluate antibacterial activity. The results are listed in Table 1. In the 3-day group, the bacterial infection rate was not different between the two types of PAC (42.86%). In the 7-day group, the bacterial infection rate of the prototype catheter (57.14%) was twice as elevated as that of the Swan-Ganz catheter (28.57%). Early in the experiment, the bacterial infection rate was as high as in the two types of PAC. The cultured bacteria were mainly normal flora in the gastrointestinal tract or the skin [14–16]. Therefore, contamination was suspected. After more carefully attending to sterilization and dressing, the bacterial infection rate decreased towards the end of the experiment.

**Table 1:** Comparison of bacterial Infection of the Swan-Ganz catheter and the prototype Catheter

GROUP	3 DAY GROUP				GROUP	7 DAY GROUP			
CATHETER	Swan-Ganz		Prototype		CATHETER	Swan-Ganz		Prototype	
EXPERIMENT NUMBER	BACTERIA	CFU/g	BACTERIA	CFU/g	EXPERIMENT NUMBER	BACTERIA	CFU/g	BACTERIA	CFU/g
1	–	0	–	0	2	*S. epidermidis*	50	S. epidermidis	50
3	S.chromogens	1	E. avium (Group D)	1	4	–	0	E. faecalis (Group D)	25
5	E. faecalis (Group D)	25	E. faecalis (Group D)	25	6	–	0	–	0
7	–	0	–	0	8	–	0	–	0
11	–	0	–	0	9	–	0	–	0
13	–	0	–	0	10	–	0	S. epidermidis	5
14	–	0	–	0	12	*S. aureus*	100	S. aureus	>100
INFECTION RATE (%)	42.86		42.86		INFECTION RATE (%)	28.57		57.14	

*CFU* Colony Forming Unit, *S. chromogens* Staphylococcus chromogens, *S. epidermidis Staphylococcus epidermidis*, *S. aureus Staphylococcus aureus*, *E. avium* Enterococcus avium, *E. faecalis* Enterococcus faecalis

Catheters implanted in pigs were isolated and examined under a microscope to determine the presence of thrombus.

The weight of thrombus was measured to evaluate antithrombotic activity from the 4th experiment. The results are listed in Table 2. In the 3-day group, the thrombus formation rate of the prototype catheter (85.71%) was twice as elevated as that of the Swan-Ganz catheter (42.85%). In the 7-day group, the thrombus formation rate was not different between the two types of PAC (71.43%). In the 3-day group, the mean thrombus amount of the prototype catheter (0.12 g) was six times as elevated as that of the Swan-Ganz catheter (0.02 g). In the 7-day group, the mean thrombus amount was 0.07 g in the prototype catheter and 0.12 g in the Swan-Ganz catheter. Despite anticoagulation therapy with LMWH, an increase in vulnerability to thrombus formation was observed in the PAC group, regardless of the type.

**Table 2:** Comparison of thrombus formation of the Swan-Ganz catheter and the prototype Catheter

GROUP	3 DAY GROUP		GROUP	7 DAY GROUP	
CATHETER	Swan-Ganz	Prototype	CATHETER	Swan-Ganz	Prototype
EXPERIMENT NUMBER	THROMBUS WEIGHT (g)	THROMBUS WEIGHT (g)	EXPERIMENT NUMBER	THROMBUS WEIGHT (g)	THROMBUS WEIGHT (g)
1	+	+	2	+	+
3	+	+	4	0.3	0.1
5	0.0	0.2	6	0.2	0.1
7	0.1	0.1	8	0.0	0.1
11	0.0	0.2	9	0.1	0.0
13	0.0	0.1	10	0.1	0.1
14	0.0	0.0	12	0.0	0.0
MEAN THROMBUS WEIGHT (g)	0.02	0.12	MEAN THROMBUS WEIGHT (g)	0.12	0.07
THROMBUS RATE (%)	42.85	85.71	THROMBUS RATE (%)	71.43	71.43

## Conclusion

In conclusion, after the 7-day implantation, there were differences in the rate of infection and thrombus formation between the Swan-Ganz catheter and the prototype catheter. The differences in these outcomes based on the material of the catheter.

## Abbreviations

CFU: Colony forming unit
E. avium: Enterococcus avium
E. faecalis: Enterococcus faecalis
LMWH: Low molecular weight heparin
PAC: Pulmonary artery catheter
*S. aureus*: *Staphylococcus aureus*
S. chromogens: Staphylococcus chromogens
*S. epidermidis*: *Staphylococcus epidermidis*
